# Prospective study of functional outcomes and return to sports after anterior cruciate ligament reconstruction in the knee

**DOI:** 10.1007/s00264-023-05973-w

**Published:** 2023-09-13

**Authors:** E. Laboute, E. James-Belin, O. Ucay, A. Caubere, E. Verhaeghe

**Affiliations:** 1 C.E.R.S., Groupe Ramsay Santé, 83 Av Maréchal de Lattre de Tassigny, 40130 Capbreton, France; 2 Service de Chirurgie Orthopédique Et Traumatologie, Hôpital d’Instruction Des Armées Sainte-Anne, 2, Bd Sainte Anne, BP 600, 83800, Cedex 9 Toulon, France

**Keywords:** Knee, Anterior cruciate ligament, Return to sport, Sport, Athletes, Factors

## Abstract

**Purpose:**

Individual factors of low rates of return to sport after anterior cruciate ligament (ACL) reconstruction were unclear. We evaluated the impact of various individual factors after ACL reconstruction for return to sport in athletes.

**Methods:**

A prospective study was performed in 1274 athletes, who had undergone ACL autograft reconstruction. Individual factors survey about return to sport was performed during the second year after surgery. Athlete responses were analyzed with a multivariate logistic model adjusted for baseline patient characteristics and an adjusted Cox model.

**Results:**

Younger age and involvement in higher-level sporting activities were associated with a significantly higher frequency and a significantly shorter time to return to sport (running, training, competition; *p* = 0.001 to 0.028). Men returned to sport more rapidly than women, for both training (*p* = 0.007) and competition (*p* = 0.042). Although there was no difference to return to sport between hamstring (HT) and patellar tendon (PT) autograft. We note that MacFL surgery (Mac Intosh modified with intra- and extra-articular autografts used the tensor fasciae latae muscle) was associated with a higher frequency (*p* = 0.03) and rapidity (*p* = 0.025) of return to training than HT. Sports people practicing no weight-bearing sports returned to training (*p* < 0.001) and competition (*p* < 0.001) more rapidly than other sports people. By contrast, the practicing pivoting sports with contact started running again sooner (*p* < 0.001).

**Conclusion:**

Younger age, male sex, higher level of sports, sportspeople practicing no weight-bearing sports, and MacFL surgery reduce time to return to sport after ACL reconstruction.

## Introduction

In the USA, the reported annual incidence of ACL reconstruction is 36.9 per 100,000 people [1]. Currently, two main reconstruction techniques are used: patellar tendon and hamstring autografts. Nowadays, using hamstring autografts was the gold standard for ACL reconstruction [2, 3]. As reported in Denmark, the proportion of hamstring autografts increased from 68% in 2005 to 85% in 2011 [4]. However, very few differences [5–7] have been clearly demonstrated between these two reconstruction procedures [8, 9]. Patellar tendon autografts are more frequently associated with anterior knee pain [8, 10], whereas hamstring autografts tend to progress toward greater residual laxity [8, 11]. Alternative graft options exist, but very few comparative studies have been performed on it. These alternatives include, in particular, hamstring autografts using only the semitendinosus (ST) with two tunnels (a femoral and a tibial tunnel) [12–14], hamstring autografts with an anterolateral graft tensor (HT + AL) with two tunnels (a femoral and a tibial tunnel) [15, 16], muscle hamstring autografts with double bundle and four anatomic tunnels (2HT) [17], and intra- and extra-articular autografts with the tensor fasciae latae muscle (MacFL) [18, 19]. Furthermore, increasing numbers of studies are focusing on the return to sport [20–22], but these studies do not sufficiently take into account morphotype data that may also have an impact on the return to sport [23–28]. No study specifies the frequencies and times to each chronological stage of the return to sport, in the same series, for a population of sportspeople practicing at different levels with multivariate analyses and adjustment for baseline patient characteristics. We therefore tested the hypothesis that baseline factors (age, sex, type and level of sport, and type of surgery) could influence return to sport.

With this prospective study, we proposed to investigate the influence of these factors on the frequency and time to return to sport after initial ligament reconstruction in a population of athletes.

## Material and methods

This was a prospective study that included athletes who had undergone a first ACL autograft reconstruction between 01/06/2017 and 31/12/2018. The study was approved by a scientific ethics committee (*Groupement de Cooperation Sanitaire Ramsay Générale de Santé pour l’Enseignement et la Recherche*, Paris, IRB N. COS-RGDS-2015–09-018), and all patients signed an informed consent form for participation.

Patients with osteotomy, bone fracture or chondroplasty, an associated medial/lateral ligament surgery, and iso or contralateral rupture were not included. When athletes were included, all their data were input into a computerized database that included complete surgical, medical, and sports-related data. All surgical information were completely determined. Patients were considered eligible for this study if they had undergone one of six different types of surgery [29] for a first reconstruction and aged more than 16 years. All the surgeries were performed by French LCA specialist surgeons. The use of arthroscopy was referred to in all the surgeries. The first surgery used the patellar tendon autografts (PT), involving transplantation of the patellar tendon (bone-patellar tendon-bone) with a single bundle, and two tunnels (a femoral and a tibial tunnel) [8]. The type of femoral and tibial fixations were interference screws. The second surgery was hamstring autografts (HT) requiring two hamstring muscles (semitendinosus and gracilis), folded over, with a single bundle and two tunnels (a femoral and a tibial tunnel) [8]. The single bundle was composed of four strands. The type of femoral and tibial fixations were endobuttons or screws. The third surgery was hamstring autografts using only the semitendinosus (ST) with a single bundle and two tunnels (a femoral and a tibial tunnel) [12–14]. The single bundle was composed of three strands. The type of femoral and tibial fixations were endobuttons or screws. The fourth was hamstring autografts with an anterolateral graft (HT + AL) with two tunnels (a femoral and a tibial tunnel) [15, 16]. It is a graft combined with anterolateral ligament reconstruction. The type of femoral and tibial fixations were endobuttons or screws. The fifth was hamstring muscle autografts with a double bundle and four anatomic tunnels (2HT) [17]. The anterior cruciate ligament consists of at least two distinct functional bundles: the anteromedial bundle and the posterolateral bundle. An anatomic double-bundle ACL reconstruction was therefore close to the native anatomy to increase the rotational stability. The sixth was the Mac Intosh modified with intra- and extra-articular autografts used the tensor fasciae latae muscle (MacFL) [18, 19]. The graft was realized with a combined internal and external anterior cruciate ligament reconstruction with the iliotibial band autograft.

Rehabilitation [25] was based on post-operative recovery for articular extension at 0° and articular flexion at more than 120°, quadriceps contraction against gravity, and techniques for walking without assistance from three to six weeks after surgery. A brace was worn for three to six weeks, as decided by the surgeon. Cardiovascular activity on a bicycle, step machine, or rowing machine was introduced progressively, and swimming (crawl) was also introduced during this period. A return to running was introduced around the third or fourth month, at the decision of the surgeon. Return to the original activity was subject to the surgeon’s approval.

Patients were contacted by telephone during the second year after surgery. Data regarding return to sport (running, training, competitive sport, same level of competition) and the time to each of these events were collected. Sports were analyzed according to discipline and were grouped according to the Arpège classification [30]. For patients playing competitively, sport level was classified as regional, national, or international, whereas patients playing non-competitively were classified as recreational athletes, such as as sports teacher, coach, or monitor.

### Statistical analyses

Multivariate logistic regression analyses were performed to evaluate the effects of the various factors on the frequency of return to sport outcome (primary endpoint), with adjustment for baseline patient characteristics. A Cox multivariate model accounting for the same factors was performed on the time to return to sport, to establish the robustness of the results in terms of the primary endpoint. The alpha risk was fixed at 5%. Statistical analyses were performed with SAS® for Windows (Version 9.4, SAS Institute Inc., Cary, NC, USA).

## Results

**Fig. 1:**
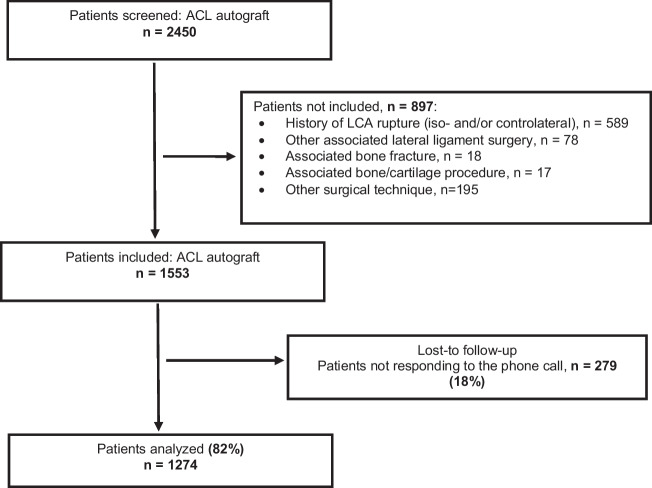
Flowchart summarizing the study design

In total, we screened 2450 athletes undergoing ACL autograft reconstruction. Of the initial population, 897 did not meet the inclusion criteria (Fig. 1). Responses (82%) from 1274 athletes were thus analyzed. The mean time between initial surgery and questionnaire completion was 19.5 months (± 4.2).

Population were analyzed for the six types of surgery (Table 1): 56% (*N* = 712) for the hamstring group (HT), 19% (*N* = 243) for the patellar tendon autograft group (PT), 10.7% (*N* = 137) for hamstring autografts using only the semitendinosus (ST), 4.1% (*N* = 52) for hamstring autografts with an anterolateral graft tensor fasciae latae (HT + AL), 6% (*N* = 76) for hamstring muscle autografts with a double bundle and four anatomic tunnels (2HT), and 4.2% (*N* = 54) for autografts with the tensor fasciae latae muscle (MacFL). Mean age was 26.2 years. The most common sport practiced was rugby (33.7%), followed by soccer, handball, and skiing.

**Table 1:** Characteristics of patients (*N* = 1274)

Variable		All
Age at surgery	*n*	1274
	Mean (standard deviation)	26.2 (7.2)
Age group	*n*	1274
	Age < = 20	231 (18.1%)
	Age 20 to 25	457 (35.9%)
	Age 25 to 30	310 (24.3%)
	Age 30 to 35	149 (11.7%)
	Age > 35	127 (10.0%)
Sex	*n*	1274
	M	933 (73.2%)
	W	341 (26.8%)
Sport	*n*	1274
	Basketball	76 (6.0%)
	Soccer	205 (16.1%)
	Handball	144 (11.3%)
	Motocross	39 (3.1%)
	Rugby	429 (33.7%)
	Ski	106 (8.3%)
	Fight sports	71 (5.6%)
	Racket sports	57 (4.5%)
	Other	147 (11.5%)
Type of sport	*n*	1274
	Weight-bearing without pivoting	51 (4.0%)
	Pivoting non-contact sans contact	269 (21.1%)
	Pivoting with contact	940 (73.8%)
	Non-weight-bearing	14 (1.1%)
Level	*n*	1274
	International	38 (3.0%)
	National	453 (35.6%)
	Regional	588 (46.2%)
	Recreational	195 (15.3%)
Surgery	HT	712 (56%)
	PT	243 (19%)
	MacFL	54 (4.2%)
	2HT	76 (6%)
	HT + AL	52 (4.1%)
	ST	137 (10.7%)

Classification ARPEGE [10]: Pivoting contact: Soccer, rugby, basketball, handball, American football, ice hockey, combat sports, fencing, bullfighting. Pivoting without contact: volleyball, racket sports, ice skating, dance, gymnastics, downhill skiing, water skiing, snowboard, surf, sailing, golf, motocross, rock, climbing, skate-board. Weight-bearing without pivoting: running, athletics, horse riding, mountain guide, bowling, cycling, shooting. Non-weight-bearing: kayaking, swimming, diving, rowing, underwater, hockey, water-polo

### Influence of baseline factors on the return to sport outcome

A return to running was reported for 94.2% of patients, a return to training for 84.8%, a return to competition for 74.3%, and a return to the same level of competition for 63.4%. The mean times for the return to sport were 4.8 months for the return to running, 7.9 months for the return to training, 9.5 months for the return to competition, and 10.5 months for the return to the same level of competition (Table 2).

**Table 2:** Frequence and time of return to running, training, competition, and same level

Factors	Variable	Frequence (%)	Time to running Months: mean, (standard deviation)	Frequence (%)	Time to training Months: mean, (standard deviation)	Frequence (%)	Competition Months: mean, (standard deviation)	Frequence (%)	Same level Months: mean, (standard deviation)
Surgery	All HT 2HT HT + AL ST MacFL PT	94.2 92.8 89.3 96.2 94.7 96.2 91.6	4.8 (2.3) 4.8 (2.3) 5.0 (2.2) 5.3 (3.3) 4.7 (2.2) 4.3 (1.7) 4.7 (2.2)	84.8 80.5 85.3 92.3 91.1 92.3 85.8	7.9 (2.6) 7.9 (2.6) 7.4 (2.0) 8.1 (3.2) 7.9 (3.2) 7.3 (2.5) 8.1 (2.6)	74.3 70.9 67.2 75 80.3 76 77.9	9.5 (2.8) 9.5 (2.7) 9.4 (2.8) 9.6 (3.5) 9.3 (2.7) 7.9 (2.2) 9.9 (3.1)	63.4 60.6 52.5 64.7 63.4 63.3 64.3	10.5 (3.0) 10.5 (3.0) 10.3 (3.1) 10.5 (3.6) 10.4 (3.1) 9.1 (2.7) 10.7 (3.0)
Sex	M W	94.4 93.6	4.7 (2.2) 4.9 (2.5)	85.3 83.2	7.8 (2.5) 8.1 (2.9)	74.8 72.6	9.4 (2.7) 9.8 (3.0)	63.2 64.1	10.3 (2.8) 10.9 (3.5)
Level									
	International National Regional Recreational	100 97.0 95.1 84.5	4.6 (2.1) 4.5 (1.8) 4.9 (2.4) 5.4 (2.9)	94.7 89.5 80.8 83.8	6.9 (2.1) 7.6 (2.4) 8.2 (2.8) 7.7 (2.5)	89.5 81.7 70.9 60.7	8.9 (3.0) 9.3 (2.8) 9.8 (2.9) 9.3 (2.6)	80.5 70.8 59.4 51.8	10.2 (3.7) 10.2 (2.9) 10.7 (3.0) 10.7 (2.8)
Type of sport	Weight-bearing without pivoting Pivoting without contact Pivoting with contact No weight-bearing	82.2 87.8 96.9 71.4	4.6 (1.9) 5.6 (3.0) 4.6 (2.0) 4.4 (1.2)	85.3 86.9 83.9 100	6.4 (3.5) 7.7 (2.6) 8.0 (2.5) 5.2 (1.7)	75 69.6 75.1 100	8.2 (3.4) 9.4 (3.0) 9.6 (2.7) 7.2 (1.6)	58.8 58.4 64.5 90	8.9 (3.0) 10.3 (3.0) 10.6 (3.0) 10.3 (4.6)

Various factors (age, sex, type of sport and type of surgery) affected return to sport, but in different ways, depending on the chronology of the steps in the return to sport.

### Age

A younger age (< 25 years versus > 25 years) was associated with a significantly higher frequency of return to sport (Table 3): for running (*p* = 0.006), training (*p* = 0.026), competition (*p* = 0.008), and return to the same level of competition (*p* = 0.005). Being younger was also associated with a shorter time to return to sport (Table 4): for running (*p* = 0.002), training (*p* = 0.076), competition (*p* = 0.028), and return to the same level of competition (*p* = 0.015).

**Table 3:** Multivariate model for the frequence of return to running, training, competition, and same level

Variable	Comparison	*p*-value	Running OR multivariable IC 95% (*N* = 1255)	*p*-value	Training OR multivariable IC 95% (*N* = 1237)	*p*-value	Competition OR multivariable IC 95% (*N* = 1163)	*p*-value	Same level OR multivariable IC 95% (*N* = 1131)
Surgery	HT vs MacFL	0.828	0.656 (0.103, 2.315)	0.030*	0.380 (0.112, 0.967)	0.433	0.898 (0.435, 1.737)	0.987	1.000 (0.532, 1.831)
Age group	Age > 25 vs. age < = 25	0.006*	0.481 (0.283, 0.8)	0.026*	0.694 (0.502, 0.958)	0.008*	0.694 (0.529, 0.911)	0.005*	0.700 (0.546, 0.899)
Level		0.010*		0.007*		< 0.001*		< 0.001*	
	International vs. Recreational		3.250 (0.87, 21.24)		4.681 (1.297, 30.093)		5.817 (2.147, 20.434)		3.375 (1.481, 8.478)
	International vs. National		1.255 (0.331, 8.266)		2.476 (0.71, 15.667)		2.105 (0.803, 7.244)		1.705 (0.785, 4.128)
	International vs. Regional		1.457 (0.391, 9.524)		4.129 (1.2, 25.989)		3.649 (1.4, 12.514)		2.636 (1.213, 6.38)
	Recreational vs. National		0.386 (0.204, 0.721)		0.529 (0.319, 0.885)		0.362 (0.237, 0.554)		0.505 (0.338, 0.756)
	Recreational vs. Regional		0.448 (0.251, 0.8)		0.882 (0.556, 1.422)		0.627 (0.421, 0.938)		0.781 (0.528, 1.156)
	National vs. Regional		1.161 (0.649, 2.116)		1.668 (1.159, 2.427)		1.734 (1.278, 2.365)		1.545 (1.178, 2.033)
Sex	W vs. M	0.335	0.784 (0.482, 1.298)	0.051	0.702 (0.494, 1.006)	0.174	0.808 (0.595, 1.101)	0.925	0.986 (0.744, 1.312)
Type of sport		< 0.001*		0.079		0.893		0.168	
	Weight-bearing without pivoting vs. pivoting without contact		0.616 (0.283, 1.414)		NS		NS		NS
	Weight-bearing without pivoting vs. pivoting with contact		0.244 (0.109, 0.577)		NS		NS		NS
	Weight-bearing without pivoting vs. no weight-bearing		1.353 (0.307, 5.294)		NS		NS		NS
	Pivoting without vs. with contact		0.396 (0.228, 0.691)		NS		NS		NS
	Pivoting without contact vs. no weight-bearing		2.194 (0.555, 7.343)		NS		NS		NS
	Pivoting with contact vs. No weight-bearing		5.540 (1.383, 18.672)		NS		NS		NS

*NS* non significative, significative (*p* < 0.05)*

**Table 4:** Adjusted multivariate analysis of the time to return to running, training, competition, and same level

Variable	Comparison	*p*-value	Running OR multivariable IC 95% (*N* = 1124)	*p*-value	Training OR multivariable IC 95% (*N* = 1179)	*p*-value	Competition OR multivariable IC 95% (*N* = 1163)	*p*-value	Same level OR multivariable IC 95% (*N* = 1131)
Surgery	HT vs MacFL	0.828	NS	0.025*	0.679 (0.504, 0.937)	0.557	0.768 (0.551, 1.106)	0.988	0.863 (0.607, 1.275)
Age group	Age < = 25 vs. age > 25	0.002*	1.224 (1.075, 1.395)	0.076	1.129 (0.988, 1.291)	0.028*	1.175 (1.018, 1.357)	0.015*	1.224 (1.041, 1.441)
Sex	W vs. M	0.227	0.913 (0.787, 1.056)	0.007*	0.813 (0.699, 0.943)	0.042*	0.845 (0.717, 0.992)	0.533	0.945 (0.79, 1.126)
Level		0.018*		< 0.001*		< 0.001*		< 0.001*	
	International vs. Recreational		1.451 (0.968, 2.113)		2.069 (1.382, 3.018)		2.527 (1.648, 3.792)		2.213 (1.382, 3.459)
	International vs. National		1.038 (0.712, 1.463)		1.510 (1.038, 2.126)		1.427 (0.977, 2.014)		1.327 (0.877, 1.93)
	International vs. Regional		1.148 (0.787, 1.617)		1.950 (1.338, 2.751)		1.943 (1.327, 2.75)		1.762 (1.16, 2.573)
	Recreational vs. National		0.716 (0.576, 0.885)		0.730 (0.586, 0.903)		0.565 (0.434, 0.726)		0.600 (0.448, 0.791)
	Recreational vs. Regional		0.792 (0.64, 0.974)		0.943 (0.761, 1.16)		0.769 (0.592, 0.986)		0.796 (0.595, 1.05)
	National vs. Regional		1.106 (0.962, 1.27)		1.291 (1.119, 1.489)		1.362 (1.171, 1.582)		1.328 (1.123, 1.569)
Type of sport		< 0.001*		< 0.001*		< 0.001*		0.06	
	Weight-bearing without pivoting vs. pivoting without contact		0.953 (0.647, 1.363)		1.065 (0.721, 1.526)		1.293 (0.83, 1.936)		NS
	Weight-bearing without pivoting vs. pivoting with contact		0.685 (0.471, 0.963)		1.317 (0.902, 1.857)		1.249 (0.816, 1.828)		NS
	Weight-bearing without pivoting vs. non-weight-bearing		1.173 (0.582, 2.621)		0.210 (0.111, 0.427)		0.226 (0.109, 0.514)		NS
	Pivoting without vs. with contact		0.719 (0.599, 0.858)		1.237 (1.036, 1.469)		0.966 (0.791, 1.173)		NS
	Pivoting without contact vs. non-weight-bearing		1.230 (0.667, 2.6)		0.198 (0.114, 0.377)		0.175 (0.093, 0.373)		NS
	Pivoting with contact vs. Non-weight-bearing		1.712 (0.936, 3.596)		0.160 (0.093, 0.304)		0.181 (0.098, 0.382)		NS

*NS* non significative, significative (*p* < 0.05)*

### Sex

Women returned to sport significantly less rapidly than men, for training (*p* = 0.007) and competition (*p* = 0.042) (Table 4).

### Sport level

Higher sport levels (Table 3) were associated with a significantly higher frequency of return to sport: for running (*p* = 0.01), training (*p* = 0.007), competition (*p* < 0.001), and return to the same level of sport (*p* = 0.001). Being higher sport level was associated with more rapidly return to sport (Table 4): for running (*p* = 0.018), training (*p* < 0.001), competition (*p* < 0.001), and return to the same level of competition (*p* < 0.001).

### Type of sport

Sportspeople practicing sports involving pivoting with contact, who are used to running, starting running again sooner than those practicing other types of sport (Tables 4; *p* < 0.001). Sportspeople practicing no weight-bearing sports, which make less demands on the knee, returned to sport more rapidly (Table 4) for training (*p* < 0.001) and competition (*p* < 0.001).

### Type of surgery

MacFL surgery (Table 3) was associated with a higher frequency of (*p* = 0.03) and a faster (*p* = 0.025) return to training than HT (Table 4).

## Discussion

The most important finding of the study was that baseline factors (age, sex, level, sport, and surgery) can influence return to sport. And we have precise the frequency and the time of the steps in the return to sport which was unknown before.

We found that the frequency of return to sport was 94.2% for running, 84.8% for training, 74.3% for competition, and 63.4% for return to the same level of competition. Our results were important to have a better comprehension of return-to-sport decision. Ardern [20] precise that return to sport is a continuum with different steps: return to participation, return to sport, and return to performance. But, in general, most studies give only a part of results like competition but not the different steps of the continuum. Dingenen [31] proposed an optimized criterion-based multifactorial return-to-sport approach based on shared decision making with a layered approach within a smooth continuum with repeated athletic evaluations throughout rehabilitation followed by a gradual periodized reintegration into sport with adequate follow-up could help to guide an individual athlete toward a successful return to sport [32, 33]. Our results were a little better than the other publications [21, 34]. Ardern reported [21] a low frequency of 55% for return to the same level of competition, and Czuppon [34] found a frequency of return to sport of 50.7% in a meta-analysis. Our study is of interest because it specifies the frequencies and times to each chronological stage of the return to sport, in the same series, for a population of sportspeople practicing at different levels. The mean times recorded were 4.8 months for the return to running, 7.9 months for the return to training, 9.5 months for the return to competition, and 10.5 months for the return to competition at the same level. These times are consistent with published results for a return to the same level of competition but more detailed: 10.7 months for basketball players in the NBA [35], 10.2 months for the soccer players of the MLS [36], and 9.8 months for ice hockey players [37].

We showed here that age influences the frequency and time to return to sport, for all the chronological stages considered (running, training or competition). No study found these results in different steps of return to play. Just Arden also reported a small effect of being younger, favoring a return to preinjury level sport (effect size, 0.3) [21]. Those over the age of 25 years are 50% less likely to return to playing at their preinjury level of sport than their younger counterparts (OR, 0.5; 95% CI, 0.3–0.8) [22], and two-thirds of athletes over the age of 32 years do not return to their preinjury level [20]. NHL players injured after the age of 30 years were found to be less likely to return to play at least one full season [37], and younger French alpine skiers at the time of injury were found to be more likely to improve their performance after returning to sport [38].

In this study, we found that sex influenced the time to return to training and competition, with men returning more rapidly than women. We confirmed results of literature like Arden who found that men were about 1.5 times more likely than women to return to the preinjury level of sport (OR, 1.4; 95% CI, 1.2–1.7) and to competitive sport (OR, 1.7; 95% CI, 1.2–2.3) [21], but we proved that it was the case for training too.

We have shown that sport level influences the possibilities for returning and the time to return to sport (running, training, competition, same level), with better results for higher levels. This completed published results, as Ardern showed that elite athletes were 2.5 times more likely to return to their preinjury level (OR, 2.5; 95% CI, 2.0–3.1) and six times more likely to return to competitive sport (OR, 5.9; 95% CI, 4.6–7.5) than nonelite athletes [21]. In our study, the frequency of return to competition was 89.5% at the international level, and the frequency of return to the same level of competition was 80.5%, a value similar to that reported by Lai [27], who found, in a meta-analysis, that 83% of elite sportspeople returned to the same level of competition. And in our study, athletes returned to the same level for 70.8% for the national level, 59.4% for the regional level, and 51.8% for others.

Our results show that the type of sport influences the frequency of return to running, but also the time to the return to running, training, and competition. Participants in no weight-bearing sports returned to running less rapidly than those involved in pivoting sports. By contrast, they returned to training and competition more rapidly. Those involved in contact sports returned to training less rapidly than those participating in pivoting sports without contact. We were unable to identify any similar published studies with which to compare our results.

We found no significant difference in the rates of return to competition between HT (70.9%) and PT surgery (77.9%) (adjusted OR = 0.718; 95% CI (0.50;1.02)), consistent with most published findings [24, 39]. By contrast, the frequency and time to return to training were significantly higher for MacFL (92.3% and 7.3 months, respectively) in our study after adjustment for the various factors in the multivariate analysis, but we were unable to identify any similar published studies. It would be interesting to confirm these results in larger population and would be important information for high-level sports people.

## Limitations of the study

We found several biases of inclusion as in all prospective studies. The different surgeons operating on the patients are a potential source of bias worthy of inclusion in analyses. Nonetheless, any associated bias was limited, given that the inclusion of patients was nationwide and there was a large number of participating surgeons, all of whom are specialists in knee reconstruction. Randomization was not performed at inclusion, but the large population, similar baseline characteristics, and adjusted analyses would have reduced potential bias. As the adjusted comparisons take into account confounding factors, they are thus interpretable with good quality.

For this study, a telephone questionnaire was performed a mean of 19.5 months after the autografting procedure. This is a short post-operative time, but our methodology was otherwise very similar to others [40–42], and the aim of this study was to analyze the initial chronological stage of the return to sport. Furthermore, our population included a large series of athletes, most practicing competitively, whereas most of the other reports have tended to focus on sports as leisure activities. The impact of the different sports practiced, along with their relationship to the level of sport practiced, was evaluated.

## Conclusion

The factors considered (age, sex, type and level of sport, and type of surgery) influence the outcome of anterior cruciate ligament reconstruction. For the return to sport, younger athletes were more likely to return to sport but did so more rapidly. Higher sport levels were associated with a higher frequency of return to sport and a faster return. Men also returned to sport more rapidly than women. Although there is no difference in the principal surgery (HT and PT), it is interessant to observe that MacFL led to a higher frequency of and a faster return to training than HT. Sportspeople practicing non-weight-bearing sports returned to training and competition more rapidly than those practicing weight-bearing sports. However, those practicing pivoting contact sports returned more rapidly to running. Finally, a young man practicing weight-bearing sports with MacFL autograft surgery is the patient with the most important frequency and faster return to sport.
